# Understanding patterns and processes in models of trophic cascades

**DOI:** 10.1111/ele.12200

**Published:** 2013-10-27

**Authors:** Michael R Heath, Douglas C Speirs, John H Steele, Kevin Lafferty

**Affiliations:** 1 Department of Mathematics and Statistics, University of StrathclydeLivingstone Tower, Glasgow, G1 1XP, UK; 2 Marine Policy Centre, Woods Hole Oceanographic InstitutionWoods Hole, MA, 02543, USA

**Keywords:** Bottom-up, density dependence, food chain, food web, harvesting, model, predator–prey, simulation, top-down

## Abstract

Climate fluctuations and human exploitation are causing global changes in nutrient enrichment of terrestrial and aquatic ecosystems and declining abundances of apex predators. The resulting trophic cascades have had profound effects on food webs, leading to significant economic and societal consequences. However, the strength of cascades–that is the extent to which a disturbance is diminished as it propagates through a food web–varies widely between ecosystems, and there is no formal theory as to why this should be so. Some food chain models reproduce cascade effects seen in nature, but to what extent is this dependent on their formulation? We show that inclusion of processes represented mathematically as density-dependent regulation of either consumer uptake or mortality rates is necessary for the generation of realistic ‘top-down’ cascades in simple food chain models. Realistically modelled ‘bottom-up’ cascades, caused by changing nutrient input, are also dependent on the inclusion of density dependence, but especially on mortality regulation as a caricature of, e.g. disease and parasite dynamics or intraguild predation. We show that our conclusions, based on simple food chains, transfer to a more complex marine food web model in which cascades are induced by varying river nutrient inputs or fish harvesting rates.

## Introduction

Worldwide impacts of the losses of top predator fauna from terrestrial and aquatic ecosystems, largely as a result of human activity, have penetrated deep into food webs, affecting vegetation cover, biogeochemistry, disease, erosion and hydrological cycles (Estes *et al*. 2011). These impacts occur because of the connectivity between taxa in food webs, so that changes in the abundance at the highest trophic levels are transferred down food chains to impact even plants and microbes. This is the phenomenon which has become known as a ‘trophic cascade’ (Carpenter *et al*. 1985; Pace *et al*. 1999; Polis *et al*. 2000). Cascades are said to be ‘top-down’ if initiated by a change in top predators, but can also be ‘bottom-up’ if initiated by a change in basal resources such as the nutrient supply to primary producers (Kagata & Ohgushi 2006).

Although trophic cascades appear to be a ubiquitous property of food chains and webs, their strength, measured as the emergent change in abundance of a given component of a food chain or web relative to a forced change elsewhere in the system, is extremely variable between ecosystems (Polis *et al*. 2000; Shurin *et al*. 2002). In some systems, cascades have been shown to extend over many trophic levels, whilst in others the observable impacts have been dissipated within one trophic level. Empirical research indicates that food chain length is important (Borer *et al*. 2005), but also that the processes governing propagation of the effects between trophic levels depend on a wide range of other factors, like behavioural interactions, disease and parasite transmission, species richness, competition for space and interference between individuals (Borer *et al*. 2005, 2006; Hammerschlag & Trussell 2011).

Statistical analyses of empirical evidence (e.g. Schmitz *et al*. 2004), and mathematical analyses of simple food chain models (e.g. Herendeen 1995; McCann *et al*. 1998b; Oksanen *et al*. 1981) have so far failed to yield a general theory that explains variation in the strength of trophic cascades. Here, we extend existing mathematical analyses of food chain formulations to reveal how density-dependent and consumption processes lead to different types and strengths of trophic cascades. We illustrate our analysis with examples from the marine realm, because depletion of predatory fish by fishing has notoriously precipitated trophic cascades in continental shelf and ocean ecosystems (Christensen *et al*. 2003; Frank *et al*. 2007; Micheli 1999).

## Brief Synopsis of Cascade Processes

**Figure 1:**
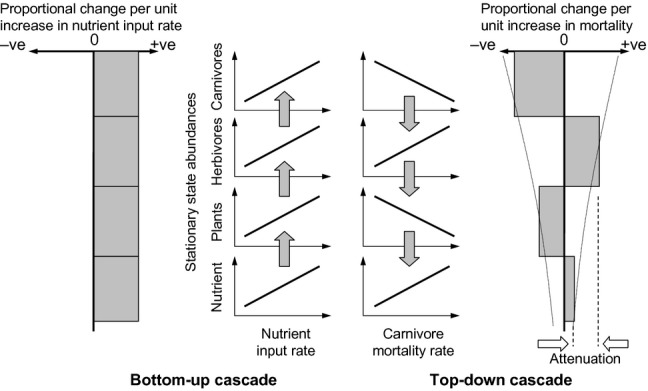
Schematic representation of the signatures of bottom-up and top-down forced trophic cascades, showing the archetypal conceptual patterns of attenuation and correlations between adjacent trophic levels based on empirical observations.

A top-down cascade is caused by a change in some factor(s) affecting the survival or productivity of the upper trophic level(s) of a food chain or web, and manifests as an inverse changes in abundance or biomass between adjacent pairs of trophic levels (Carpenter *et al*. 1985; Pace *et al*. 1999). Hence, for a three-level food chain, a decrease in the abundance of top predators results in higher abundances of mid-level consumers and lower abundance of basal producers, though the effect is often progressively attenuated with transfer between successive levels (Fig.1). Some of the clearest such signatures of top-down cascades in the natural world arise from aquatic systems (Strong 1992), especially those which have been heavily exploited by fisheries (Baum & Worm 2009). Data from many marine ecosystems have been interpreted as indicative of trophic cascades (Sala *et al*. 1998). Three notable examples that show the effects of fishing propagating down the trophic levels from piscivorous fish to plankton and nutrients are listed in Table1.

**Table 1:** Categories used to demonstrate trophic cascades in three case studies: the Eastern Scotian Shelf (Frank *et al*. 2005), Black Sea (Daskalov 2002) and the Baltic (Casini *et al*. 2008)

Trophic level	Eastern Scotian shelf	Black Sea	Baltic
4	Benthic fish	Predatory fish	Cod
3	Small Pelagic fish/Benthic invertebrates	Planktivorous fish/gelatinous zooplankton	Sprat
2	Herbivorous zooplankton	Crustacean zooplankton	Zooplankton
1	Colour Index (CPR)	Phytoplankton	Chlorophyll
0	Nitrate	–	Oxygen

A bottom-up cascade (Kagata & Ohgushi 2006) occurs when a change in nutrient supply leads to similar changes in equilibrium abundances at all trophic levels, at least until abundances are constrained by other factors. In the marine realm, El Niño events in the eastern Pacific Ocean reduce the upwelling of nutrient, leading to large (> 50%) decreases in abundance across all trophic levels of the upper ocean pelagic system (Barber & Chavez 1983; Cury *et al*. 2000). Fluctuations in primary production are reflected in zooplankton, fish and higher trophic level abundance across a range of shelf sea ecosystems (Frank *et al*. 2006; Frederiksen *et al*. 2006). However, meta-analyses of data from experimental nutrient-enrichments of aquatic mesocosms show that enhanced primary production is often not uniformly transferred up the food chain but may skip alternate levels, so that increased phytoplankton production leads to small or no change in zooplankton, but increased yield of planktivorous fish (Borer *et al*. 2006; Brett & Goldman 1997). These differences in the propagation of a bottom-up cascades ought to be explainable in terms of biological processes.

Trophic cascade concepts have their origins in the ‘Green World’ hypothesis of Hairston *et al*. (1960). This states that under natural conditions ‘herbivores are seldom food-limited, appear to be predator-limited, and therefore are not likely to compete for common resources’. Proponents suggested that these processes explained the consequences for herbivore communities of removing predators–the factor limiting their growth shifts from predation to food intake, and their populations expand, precipitating overgrazing and loss of vegetation (e.g. Pace *et al*. 1999; Spiller & Schoener 1994). However, there are many contentious aspects of ‘cascade theory’, principally its reliance on the assumption of a food chain comprising functional groups–plants, herbivores, predators and parasites–rather than a web of species (Borer *et al*. 2005; Matson & Hunter 1992; Murdoch 1966; Persson 1999; Power 1992), and the effects of compartmentalisation or subwebs (Krause *et al*. 2003) which may inhibit cascades by creating weak links within the food web.

In addition to effects arising from consumption of prey, a range of behavioural responses may also be involved in determining the properties of trophic cascades (Hammerschlag & Trussell 2011; Schmitz *et al*. 2004). Many experiments have shown that ‘fear of being eaten’ is a powerful force, and the mere presence of a predator species elicits a change in behaviour and/or physiology of its prey (e.g. Brown & Kotler 2004; Wasserman & Froneman 2013) which may precipitate an indirect effect on basal resources equivalent to a cascade. This effect is referred to as a ‘trait-mediated indirect interaction’ as distinct from the consumptive or ‘density-mediated indirect interactions’ at the heart of the Green World hypothesis. Results from a wide range of manipulative experiments on small-scale ecological systems have revealed a wide range of trait-mediated non-consumptive effects in predator–prey relationships and indicated their possible roles in cascade effects (Borer *et al*. 2005; Polis *et al*. 2000; Schmitz *et al*. 2004).

Meta-analyses of field survey data have concluded that cascades are strongest in marine benthos systems and weakest, i.e. most strongly attenuated, in marine plankton and grasslands (Shurin *et al*. 2002). Data from a range of North Atlantic and other oceanic marine systems show a relationship between cascade attenuation and species richness with strongest cascades in species-poor high latitude systems (Baum & Worm 2009; Frank *et al*. 2007). However, other analyses including both aquatic and terrestrial systems have concluded that there is no general relationship between species diversity and cascade strength (Borer *et al*. 2005; O'Connor & Bruno 2009). Instead, metabolic and taxonomic properties of predator communities explain the largest part of cascade strength variability, with the strongest cascades in systems dominated by invertebrate herbivores and endothermic vertebrate predators (Borer *et al*. 2005).

## Terminology - Regulation and Forcing

The terms ‘top-down control’ and ‘bottom-up control’ have assumed common usage in relation to cascade patterns (e.g. Baum & Worm 2009; Cury & Shannon 2004; Estes *et al*. 2011; Frank *et al*. 2007; Scheffer *et al*. 2005). However, the term ‘control’ has a variety of usages in ecology and has been a source of debate since the 1950s. The usage in the phrase ‘top-down control’ refers to the role of a varying factor in exerting an influence on other components of the systems (*sensu* Hairston *et al*. 1960; Menge 1992). However, an alternative usage (*sensu* Milne 1962; Nicholson 1957) refers to mechanisms or processes within food webs, specifically self-limitation processes or density dependence, which lead to alteration in the per capita rate of change in a population as a direct function of its abundance. Clearly, these usages of ‘control’ have different meanings, and are potentially confusing for understanding of cascade dynamics.

For the purposes of our analysis, we discarded the term ‘control’. Instead, we use the term ‘regulation’ to refer to density-dependent processes within food webs, and ‘forcing’ to refer to exogenous factors which drive changes. We refer to top-level, interior-level and bottom-level regulation to indicate the trophic level in a food chain at which density dependence occurs, and bottom-up or top-down forcing to refer to type of exogenous factors affecting the system, e.g. changes in nutrient input rate at the base of a food chain, or a density-independent mortality rate applied to the top trophic level. Note that the levels at which regulation may occur and the different types of forcing, are not mutually exclusive. Indeed, in the marine context two of the main forcing factors, nutrient inputs and fishing, often act simultaneously on the lowest and highest trophic components of the ecosystem respectively.

## Building Blocks for Mathematical Models of Trophic Cascades

Here, we briefly summarise the main mathematical representations of resource consumption and density-dependent regulation in food chain models. The simplest description of resource consumption, or prey-dependent uptake response, is the classic Lotka–Volterra equation in which per capita uptake by the consumer is a direct linear function of prey abundance. Whilst this represents a well-established starting point for mathematical analysis, it is hardly realistic. In the real world, a range of biological processes lead to a variety of nonlinear responses of per capita uptake rates as prey abundance increases. The most commonly applied such relationship is the Holling Type-II equation (Holling 1959) which expresses a saturating per capita rate of uptake (g) of prey (x_1_) by a consumer (x_2_), in terms of a search rate (c) and a time for processing (b), as 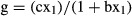
1which can be reconfigured in terms of a prey half-saturation coefficient k = 1/b, and a maximum uptake rate by the predator a = c/b 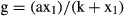
2

This form is widely used in terrestrial and aquatic food chain models (Gentleman *et al*. 2003), but there are other nonlinear formulations of prey-dependent uptake relationships which can be configured to represent e.g. the dynamic consequences of consumers switching between preference for alternative prey (e.g. Holling Type-III function): 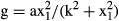
3

Neither the linear Lotka–Volterra nor the nonlinear Holling relationships include any form of regulatory mechanism and hence food chain models based solely on these forms exhibit neutral stability or instability. The introduction of regulation at either the resource or the consumer level is often achieved by a quadratic loss term to suppress responsiveness representing, e.g. competition for an un-modelled resource (e.g. space for sessile taxa), cannibalism (or intra-guild predation in the context of a model based on trophic groups), incidence of disease epidemics, or consumption by an un-modelled predator. Other authors have referred to this mathematical process as ‘interference’ (McCann *et al*. 1998b; Polis & Hort 1992). In the context of a Lotka–Volterra predator–prey couplet this can be represented as follows: 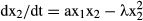
4

where λ = consumer quadratic mortality parameter. The intensity of density-dependent regulation increases with λ.

In contrast to mortality regulation, the introduction of consumer-density dependence of per capita uptake rate suppresses responsiveness by regulating the flux between prey and consumers. The simplest form of uptake regulation is referred to as ratio dependence (Arditi & Ginzburg 1989, 2012). In the context of an underpinning linear Lotka–Volterra prey dependency: 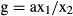
5

The ratio-dependent uptake formulation was intended to represent sharing of resources, behavioural interference between consumers to their mutual impairment, enhanced escape reactions by prey, sheltering in refuges with increasing predator density (Hill & Weissburg 2013), or the foraging of predators in a patchy prey environment (Anderson 2010; Cosner *et al*. 1999). Hence, ratio-dependence is conceptually a mathematical caricature of a trait-mediated response (Arditi & Ginzburg 2012). There are many observational and experimental examples of top-down forced prey behavioural responses to predators of this type, with evidence that they lead to impacts on basal resources–and hence a *de facto* cascade effect (Griffin *et al*. 2011; Schmitz *et al*. 2004; Trussell *et al*. 2006). However, the ratio-dependent formulation has been criticised for a variety of reasons, but mainly because it implies the extreme property that per capita uptake rate tends to infinity as consumer abundance tends to zero (Abrams 1994). A variant of ratio-dependence which alleviates this property is the so-called foraging arena scheme (Walters *et al*. 2000). Again, in the context of an underpinning linear Lotka–Volterra prey dependency: 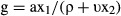
6

where a/ρ = maximum per-capita uptake rate by x_2_, and the x_2_ density at half maximum per capita uptake rate is given by ρ_/_υ. Hence, the intensity of density-dependent regulation increases with υ and if υ = 0 then uptake conforms to Lotka–Volterra with no density dependence. An equivalent form conferring consumer-density dependent regulation on the Type-II uptake function is the Beddington-DeAngelis equation (Beddington 1975; DeAngelis *et al*. 1975): 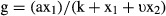
7

The above mathematical functions constitute the main building blocks for constructing food chain models, but there remains the issue of the dynamics at the lowest trophic level. A common solution is to assume that the lowest trophic level comprises primary producers that grow logistically in the absence of herbivores: 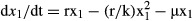
8

where r = intrinsic growth rate of x_1_, μ = proportion of x_1_ consumed per unit time by herbivores (x_2_), and k = carrying capacity for x_1_. At equilibrium, 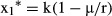
9

The logistic equation does not explicitly represent the nutrient resources on which the primary producers depend, but in reality nutrient concentrations form a key part of the top-down cascade response in at least some aquatic ecosystems (Table1), and their input rates constitute bottom-up forcing factors. Although there are at least two ways to derive eqn 8 from nutrient resource considerations, both are problematic for our current purpose. The first involves assuming that the nutrient is inexhaustible so that its concentration in the environment is constant (and hence r is constant) and that the regulation term (r/k) arises through some unspecified mortality. The second is by assuming that the nutrient available for uptake can be depleted, but that the sum of the available and consumed nutrient is constant. In this second case, eqn 8 is only valid provided x_1_ < k. Moreover, it implies that the introduction of herbivores (and higher trophic levels) will change r as they lock-up some of the available nutrients.

An alternative to logistic primary producers for the base of a food chain model is to explicitly represent a nutrient resource (x_0_) using a chemostat function (Smith & Waltman 1995). In its simplest form: 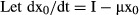
10

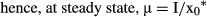
11

where, I = external input rate of x_0_, and μ = proportion of x_0_ consumed per unit time (by plants). Thus, the chemostat constitutes a density-dependent uptake regulation mechanism in its own right because the equilibrium weight-specific consumption rate of nutrient decreases with increasing nutrient mass.

## Brief Review of Previous Mathematical Analyses

Oksanen *et al*. (1981) investigated a model system comprising plants, herbivores and carnivores, linked by Type-II prey-dependent uptake relationships, with quadratic regulation of plants by virtue of assuming their growth to be logistic. The model was forced by varying the intrinsic growth rate parameter applied to the plants, and a density-independent mortality rate applied to the carnivores. This model reproduced aspects of archetypal top-down cascade effects; in particular, top-down forced decreases in carnivores produced increases in plants. Bottom-up forced changes in plant production were transferred to the carnivores without affecting the intervening herbivores, as in some experimental mesocosm studies (Borer *et al*. 2006; Brett & Goldman 1997). Generalising this model to any length of food chain, the authors showed a ‘skipped-level’ pattern of bottom-up transmission. For food chains with odd numbers of trophic levels, increases in production at the lowest level led to increased biomass at odd-numbered trophic levels but not at even. Conversely, in food chains with even numbers of levels, increased basal production led to increased biomass at even-numbered levels but not at odd. McCann *et al*. (1998b) reached a similar conclusion with an aquatic ecosystem version of the same model: an increase in phytoplankton production led to an increase in planktivorous fish but no change in zooplankton. The introduction of ratio-dependent regulation of zooplankton uptake rates altered the results such that changes in planktivorous fish no longer had any top-down effect on phytoplankton, whilst changes in phytoplankton production propagated up the food chain with omni-directional responses at all levels but with uneven sensitivity between adjacent levels. Finally, a model with density-dependent mortality regulation at the zooplankton level produced both the archetypal top-down and bottom up patterns. The authors proposed that density-dependent mortality, caricaturing intra-guild predation or disease dynamics, is a key property which determines cascade dynamics in aquatic systems.

In contrast to McCann *et al*. (1998b), Herendeen (1995) showed analytically that a model food chain with ratio-dependent uptake between all trophic levels not only reproduced the correlation signatures of a top-down cascade, but the strength could attenuate down the chain, whilst a bottom-up cascade propagated with approximately uniform strength at all levels. Subsequently, Herendeen (2004) extended the analytical approach to incorporate functional relationships which varied among levels and for which the degree of prey dependence and regulation could be varied independently. The conclusion, in line with McCann *et al*. (1998b), was that both top-down and bottom-up effects were found to depend strongly on mortality regulation, whilst bottom-up effects were additionally dependent on the form of the dependency of uptake on prey density.

An alternative approach to modelling trophic cascades involves size spectrum simulations (Andersen & Pedersen 2010; Rossberg 2012). Models of this type have been especially applied to marine ecosystems. Here, the community is composed of traits (asymptotic body size) rather than species, and each trait is represented by a separate spectrum of body size. Within a trait, progression between size classes represents growth. Mortality arises from predation and background sources, and closure of the life cycle by recruitment. The key process in the model is prey selection, which is controversially assumed to be solely on the basis of the size difference between predator and prey rather than on traits (see review in Rossberg 2012). Analysis shows that imposition of an external mortality forcing factor on size classes at the upper end of the community size range creates a damped oscillation down the spectrum towards smaller sizes, analogous to the alternating correlation signature of a top-down cascade in conventional food chain model. The degree of damping is synonymous with attenuation in a food chain. Additional external mortality at intermediate sizes, and broadening of the prey size selection interval by predators, both act to intensify damping of the size spectrum cascade. Regulation in such a model is effected by a combination of nutrient supply at the smallest end of the size spectrum, the recruitment process within each trait, and within-trait predation subject to the constraint of a given prey size selection interval. The latter process is analogous to the density-dependent mortality terms, representing intra-guild predation, in food chain models. Whilst size spectrum models clearly have many attractive features, we do not explicitly consider them further in this article.

In summary, the previous literature on mathematical analyses of trophic cascades indicates that whilst the archetypal signature of a top-down forced cascade is a reasonably robust feature of mathematical formulations of food chains and size spectra, the corresponding bottom-up cascade is not. The latter is dependent on the details of regulatory processes at individual trophic levels and the nature of uptake responses. However, the precise details remain obscure. In particular, with respect to representations of trait-mediated interactions, the analyses have been confined to ratio-dependent regulation and have not considered the less extreme forms such as foraging arena. In the following sections, we extend the findings of Herendeen (1995, 2004) and McCann *et al*. (1998b) by systematically evaluating the roles of alternative forms of density dependence in combination with linear and nonlinear uptake responses.

## Transfer Between Levels Depends on Regulation

### Linear uptake responses

The simplest description of a predator–prey couplet has an underlying linear Lotka–Volterra form. To this, we added regulation by either density-dependent mortality, or consumer-dependent uptake. These regulatory processes are not necessarily mutually exclusive and could operate jointly, but our aim here was to compare their individual properties. Hence, with regulation by density-dependent mortality: 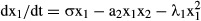
12

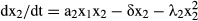
13

where δ = density-independent mortality parameter applied to the consumer, and σ = intrinsic growth rate parameter of the prey.

With regulation by consumer-dependent uptake rate: 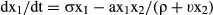
14

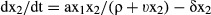
15

**Figure 2:**
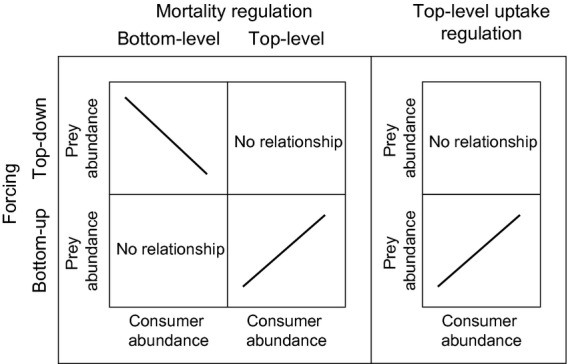
Steady state consumer vs. prey abundance responses of a simple Lotka–Volterra type system (eqns 12–15) with predator or prey density-dependent regulation. The upper row refers to top-down forcing of the density-independent mortality rate; the bottom row to bottom up forcing of prey growth rate.

The striking feature of steady state analytical solutions to eqns 12–15 (Tables S1–S2 (Supporting Information Appendix S1), and Fig.2) was that combinations of top-level regulation with top-down forcing, or bottom-level regulation with bottom-up forcing, resulted in no relationship between equilibrium abundances of prey and consumers. On the other hand, bottom-up forcing in combination with top-level regulation resulted in the expected direct relationship between prey and consumer abundances, regardless of whether regulation was implemented though mortality or uptake rates. Similarly, top-down forcing in combination with bottom-level regulation led to the expected inverse relationship between prey and consumer abundances.

We extended the simple predator–prey couplet to a food chain by (1) adding additional consumers to create a sequence of trophic levels, and (2) introducing a chemostat regulated nutrient resource at the base of the chain. We represented the nutrient resource as follows: 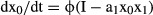
16

where x_0_(t) = nutrient concentration at time t; φ = switch parameter (0 or 1) between a static or dynamic nutrient resource; I = nutrient input rate (bottom-up forcing parameter). With quadratic mortality regulation at each subsequent trophic level: 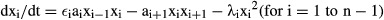
17

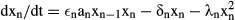
18

where x1(t) = primary producer; for 2 ≤ i ≤ n x_i_(t) = consumers, and the parameters are: a_i_ = (linear) response parameter for uptake of prey i-1 by consumer i; ε_i_ = assimilation efficiency of consumer i. Again, regulation by density-dependent mortality and uptake need not be mutually exclusive, but we chose here to represent their effects independently. Hence, the equivalent equations with consumer uptake regulation instead of density-dependent mortality were as follows: 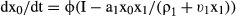
19

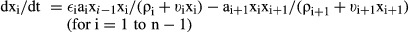
20

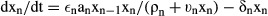
21

**Figure 3:**
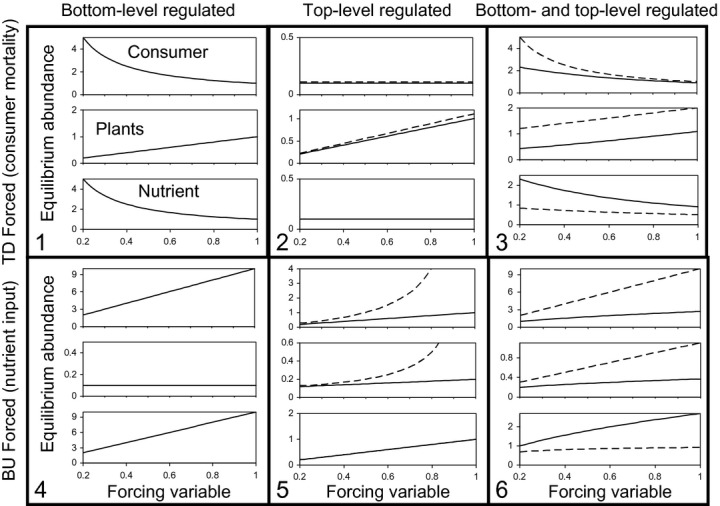
Analytical steady states of a simple food chain system with linear prey-dependency (eqns 16–21), to top-down (TD) and bottom-up (BU) forcing, depending on whether regulation is located at the bottom level (BL), top level (TL) or both, of the food chain. Parameters used in the analysis were a_i_ = 1; ε_i_ = 1, δ = 0.1 (except when treated as the forcing variable), and λ = 0.1. Solid lines for cases 2, 3, 5, and 6 show the response with top-level mortality regulation, dashed with top-level uptake regulation. See Table2 for detailed configuration of each case, and Tables S3–S4 (Appendix S1) for the analytical solutions.

By varying parameters of eqns 16–21, we could create a range of permutations of forcing and regulation. Clearly, there are many such permutations, but our aim here was to analyse independently the effects of mortality and uptake regulation under top-down and bottom-up forcing. With n = 2 (nutrient plus 2 trophic levels), we derived the analytical steady state solutions representing three model structures - bottom-level (chemostat) regulation only; top-level regulation only; both chemostat and top-level regulation acting simultaneously. Then, for each structure, we analysed two forcing scenarios [top-down (varying δn) or bottom-up (varying I)]. For each case with top-level regulation, we analysed the effects of density-dependent mortality or uptake as alternatives (Table 2, Fig.3). Stability analysis of the steady state solutions to these permutations (Appendix S1, Tables S3–S4) showed that the models which include chemostat regulation (all cases except 2 and 5) always have a single interior steady state (positive for all species), and that the steady state is asymptotically stable. With top-level mortality regulation only (cases 2 and 5) a single interior steady state exists provided that δn is sufficiently small to permit the consumer to persist, and that if this steady state exists, it is stable.

The analytical steady state solutions showed that the archetypal cascading pattern of alternating trends in abundance in response to top-down forcing, arose under both bottom-level regulation (case 1), and combined bottom and top-level regulation (case 3), but not with top-level regulation alone (case 2) (Fig.3). The only regulation scenario which supported both the archetypal top-down and bottom-up cascades was that which combined both top level and bottom level regulation (cases 3 and 6). The form of top-level regulation (mortality vs. uptake) affected the linearity of the response of trophic levels to the forcing variable, and the sensitivity at the various levels, but did not alter the conformity to the archetypal cascade responses.

### Nonlinear uptake responses

We adapted the food chain models expressed by eqns 16–21 to include the Type-II prey-dependent response with either consumer mortality or consumer uptake regulation. With mortality regulation: 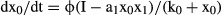
22

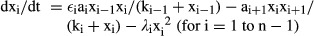
23

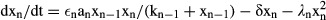
24

With consumer uptake regulation: 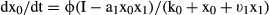
25

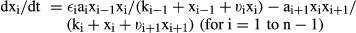
26

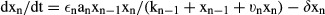
27

Using the analytical steady state solutions to eqns 22–27 for n = 2 with mortality or uptake regulation at the top-level only (see Appendix S1, Tables S5–S6), we repeated the series of case analyses listed in Table2. The steady-state responses to the top-down mortality forcing factor and the bottom-up nutrient input were directionally equivalent to the corresponding cases for the linear prey-dependent response analyses, but the range of parameters defining stable solutions was much narrower (Fig. S1 in Appendix S1). Again, the only structure which reproduced both the archetypal top-down and bottom-up cascade patterns was that which combined both bottom-level regulation and either top-level mortality regulation, or consumer uptake regulation.

**Table 2:** Case situations for analytical solutions to nutrient plus 2-level food chain models with optional chemostat regulation at the bottom level, and optional mortality or uptake regulation at the top. Equation numbers refer to the numbering in the main text. In case 5, bottom-up forcing can only be achieved by changing the constant initial nutrient concentration. Hence, in the absence of a consumer, x_1_ would increase exponentially with intrinsic rate r = ε_1_ a_1_ x_0_. In all cases, the parameters a and ε = 1. Analytical solutions to the equations based on linear uptake responses are given in Tables S3 – S4 (Appendix S1) and graphical solutions in Fig.3; the equivalent for equations based on Type-II uptake response are in Tables S5–S6 and Fig. S1 (Appendix S1)

Case	1	2.1	2.2	3.1	3.2
Linear uptake equations	16–18	16–18	19–21	16–18	19–21
Type-II uptake equations	22–24	22–24	25–27	22–24	25–27
Density dependence type	Chemostat regulation only	Top-level mortality regulation only	Top-level uptake regulation only	Both chemostat and top-level mortality regulation	Both chemostat and top-level uptake regulation
Density dependence parameters	φ = 1, λ_1_ = λ_2_ = 0	φ = 0, λ_1_ = 0, λ_2_ = 0.1	φ = 0, υ_1_ = 0, υ_2_ = 1	φ = 1, λ_1_ = 0, λ_2_ = 0.1	φ = 1, υ_1_ = 0, υ_2_ = 1
Forcing parameter	δ (top–down forcing)	δ (top–down forcing)	δ (top–down forcing)	δ (top–down forcing)	δ (top–down forcing)
	4	5.1	5.2	6.1	6.2
Linear uptake equations	16–18	16–18	19–21	16–18	19–21
Type-II uptake equations	22–24	22–24	25–27	22–24	25–27
Density dependence type	Chemostat regulation only	Top-level mortality regulation only	Top-level uptake regulation only	Both chemostat and top-level mortality regulation	Both chemostat and top-level uptake regulation
Density dependence parameters	φ = 1, λ_1_ = λ_2_ = 0	φ = 0, λ_1_ = 0, λ_2_ = 0.1	φ = 0, υ_1_ = 0, υ_2_ = 1	φ = 1, λ_1_ = 0, λ_2_ = 0.1	φ = 1, υ_1_ = 0, υ_2_ = 1
Forcing parameter	I (bottom–up forcing)	x_0_(0) (bottom–up forcing)	x_0_(0) (bottom–up forcing)	I (bottom–up forcing)	I (bottom–up forcing)

## Cascade Strength

Although the directional effects of top-down and bottom-up forcing on abundance at each level of the 3-component chain were not dependent on whether top-level regulation was effected through mortality or uptake, the manner in which the effects were transmitted between levels was fundamentally different. Cascade strength is a measure of the extent to which the effect of a top-down or bottom up forced change is transmitted through the food chain. If the signal is attenuated so that the directly forced change has a diminishing effect on remote trophic levels, then this would be said to be a weak cascade. We measured the proportional response at any given level, by comparing the equilibrium state resulting from a default value of either bottom-up or top-down forcing (I or δ_n_), with a scenario in which the forcing rates was doubled: 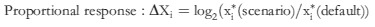
28

Here, ΔX_i_ = 0 signifies no change in trophic level i as a result of the doubling in forcing factor value, whilst a value of -1 indicates a halving, and + 1 a doubling. Cascade strength is then expressed as the differences in response between the upper and lower trophic levels: 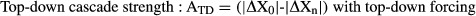
29

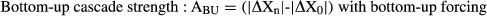
30

Here, a negative value of A indicates that the direct effect of the forcing signal is attenuated with depth or altitude through the food chain, and a positive value indicates amplification.

**Figure 4:**
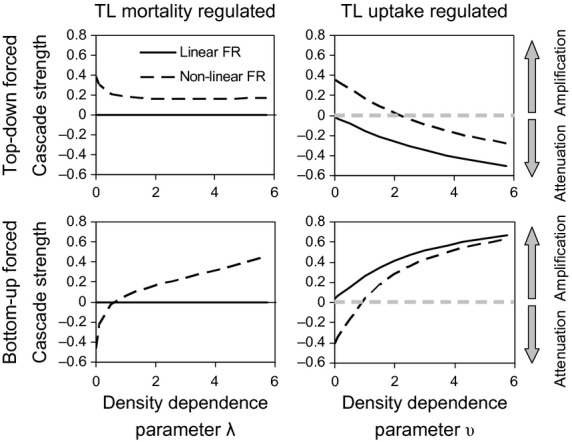
Steady-state effects of density-dependent regulation on the strength of top-down and bottom-up trophic cascades, derived from analytical solutions to the 3-level food chain equations. Positive values of the strength index indicate amplification of the top-down or bottom-up effect; negative values indicate attenuation. Solid lines show the strength of the response with a linear functional relationship (FR) between uptake and prey abundance, dashed with a nonlinear (Type-II) relationship. The *x*-axis in each case refers to values of the parameter determining the intensity of either mortality or uptake regulation. Top-down cascade strength is as specified by eqn 29, bottom-up strength by eqn 30.

The steady-state solutions for the three-level food chain model based on a linear prey-dependency with regulation at the bottom and top levels (eqn 16–21; Tables S3–S4) showed that with mortality regulation, increasing the density dependence parameter λ suppressed the response of the top-level component (x_2_) to a doubling of the top-down forcing factor (δ). The effect was then transmitted uniformly down the chain to lower levels. In contrast, with uptake regulation the density dependence parameter υ had no effect on the response of the consumer (x_2_) but suppresses the response of the resource (x_1_). Hence, top-level uptake regulation had a natural tendency to attenuate a top-down forcing signal and amplify a bottom-up forcing factor, whilst top-level mortality regulation did not (Fig.4).

Repeating the exercise with the 3-level food chains based on Type-II uptake response (eqn 22–27; Tables S5–S6) showed that with top-level mortality regulation the system had a natural tendency to amplify a top-down forcing signal (Fig.4). With top-level uptake regulation the Type-II food chain had a more complicated response to top-down and bottom-up forcing producing either amplification or attenuation, depending on the value of the density-dependent parameter υ and the rate of nutrient input.

To analyse longer food chains we set n = 3 (nutrient plus 3 trophic levels), and φ = 1 (chemostat regulation) in all cases. For mortality regulation cases, we set λ_n_ = 1 (top-level mortality regulation) but also permitted cases of λ_i<n_ = 1 to enable regulation at interior levels of the food chain. Similarly, in cases of uptake regulation we set υ_n_ = 1 (top-level uptake regulation) but also permitted cases of interior level regulation (υ_i<n_ = 1). In all cases, we solved the equations by numerical integration and output the equilibrium results after 4000 time steps (x_i_* = x_i_(t = 4000)) for default and doubled values of the forcing parameters (δ or I) so as to calculate the proportional response index ΔX_i_.

**Figure 5:**
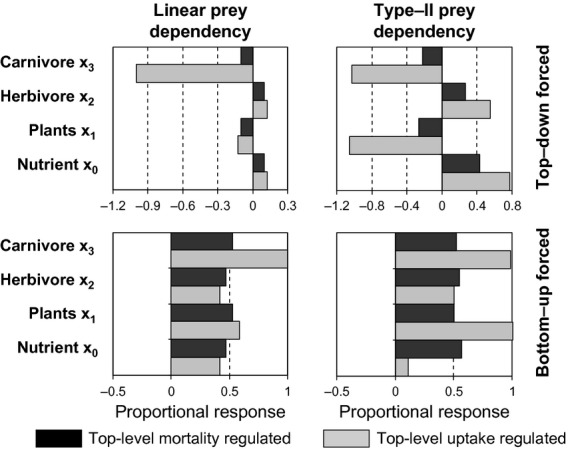
Proportional response of 4-level food chain models to a doubling of top-level forcing (density-independent mortality; top row), or bottom up forcing (nutrient input rate; bottom row), with mortality or uptake regulation only at the top-trophic level (λ_n_ = 1, or υ = 1). Left column, linear prey dependency; right column, Type-II nonlinear prey dependency. Pale shaded bars, uptake regulation; dark shaded, mortality regulation. The proportional response is calculated as in eq. 28, i.e. log_2_(abundance with doubled-forcing factor/abundance with default forcing factor).

With regulation at the top-level only (Fig.5), the results corresponded to the analytical synthesis of the three-level food chain (Fig.4). With a linear uptake response, top-down effects were transmitted uniformly through the food chain from the penultimate level down. The nonlinear Type-II uptake response led to amplification of top-down forced effects regardless of regulatory mechanism. Bottom-up forced effects were relatively uniformly transmitted up the chain with top-level mortality regulation, but unevenly with top-level uptake regulation.

Adding regulation at internal trophic levels (Fig. S2 in Appendix S1) led to attenuation of top-down forced effects throughout the chain rather than simply between the upper pair of levels in all cases except the combination of a Type-II uptake response and uptake regulation. Here, the intensity of the density dependence resulting from the given parameter values was insufficient to overcome the amplifying property on the underlying Type-II relationship. Interior mortality regulation led to more uniform transmission of bottom-up forced effects through the food chain, but interior uptake regulation did not, with strong dis-proportionality of response between adjacent trophic levels (Fig. S2 in Appendix S1).

The key points to emerge from our analyses are summarised in Table3. The first is a minimum requirement for end-member regulation of food chain models in order to reproduce the archetypal cascade patterns portrayed in Fig. 1–bottom-level regulation for a top-down cascade and top-level regulation for a bottom-up cascade. Models which lacked the appropriate end-member regulation displayed the ‘skipped-level’ pattern of cascade propagation (Fig. 3, cases 2 and 4). Various combinations of uptake relationship and types of density-dependent regulation resulted in top-down cascade attenuation reminiscent of the archetypal pattern. However, the propagation of bottom-up cascades was more complicated. Top and interior-level mortality regulation always resulted in archetypal bottom-up cascades with positively correlated responses at adjacent pairs of trophic levels. Uptake regulation led to bottom-up cascades displaying varying degrees of skipped-level propagation, especially in combination with Type-II uptake responses.

**Table 3:** Summary of the consequences for cascade strength of different combinations of uptake relationship and regulation method applied to all levels of a model food chain. The summary assumes chemostat regulation at the lowest level (nutrients) in all cases. Skipped-level transmission refers to the cases where an increase in nutrient causes no change in plants, but an increase in consumers

Uptake form	Regulation method	Top-down cascade strength	Bottom-up cascade strength
Linear	None	Uniform transmission down the chain	Skipped-level transmission, i.e. no relationship between adjacent levels. Strongly alternating amplification/attenuation between successive pairs of levels
Linear	Mortality	Attenuation down the chain	Tending to uniform transmission with increasing intensity of regulation
Linear	Uptake	Attenuation down the chain	Alternating amplification/attenuation between successive pairs of trophic levels
Type-II	None	Amplification down the chain	Skipped-level transmission, i.e. no relationship between adjacent levels. Strongly alternating amplification/attenuation between successive pairs of levels
Type-II	Mortality	Attenuation with strong regulation, amplification with weak regulation	Tending to uniform transmission with increasing intensity of regulation
Type-II	Uptake	Attenuation with strong regulation, amplification with weak regulation	Alternating amplification/attenuation between successive pairs of trophic levels

## Marine Food Web Simulations

The assumption of a food chain potentially breaks down when a web is considered because, as many authors have observed, the various components in a web no longer have integer trophic levels. Connections within the web are not only vertical but horizontal or diagonal due to omnivory, and developmental ontogeny of diet (Persson 1999; Wollrab *et al*. 2012). For example, in a species rich marine system such as Georges Bank, fish form a relatively small component (28–37%, average 1963–2002) of the diet of the so-called piscivore fish guild, with the remainder comprising plankton (18–29%) and benthos (37–45%) (Garrison & Link 2000). In addition, the overall web may comprise several partially isolated subsystems (compartments; Krause *et al*. 2003), such as under- and over-ground in the terrestrial realm, or detrital, pelagic and benthic subsystems in the aquatic (Attayde & Ripa 2008). Given this complexity, equilibration of the overall web to a change in productivity or removal rate in any one sector may not necessarily conform closely to the archetypal cascade patterns.

We examined top-down and bottom-up cascade effects in a marine food web model of intermediate complexity (Allen & Fulton 2010; Steele *et al*. 2013), which represents the fluxes of nutrient (nitrogen) through the North Sea ecosystem from dissolved inorganic to birds and mammals, and regeneration through excretion and mineralisation of detritus (Heath 2012; see Appendix S2 in Supporting Information). The model incorporates Type-II functional relationships between each predator–prey couplet in the food web, chemostat-like regulation at the lowest trophic level and density-dependent (quadratic) mortality regulation of the upper trophic levels (top levels plus most interior levels). External forcing factors include sea surface irradiance, temperature, hydrodynamic fluxes, freshwater input, river and atmospheric nitrate and ammonia inputs, ocean boundary nitrate, ammonia and suspended particulate concentrations, and density-independent fishery harvesting rates of shellfish, pelagic and demersal fish. Using simulated annealing to explore the parameter space, the stationary state of the model has been fitted to a suite of observed data from the North Sea collected between 1970 and 1999, whilst being forced by 1970–1999 average monthly external forcing data (Heath 2012). To expose cascading patterns, we compared the stationary annual mean abundances of model components in the fitted 1970–1999 simulation (hereafter referred to as the default run), with the equivalent values from scenario runs in which one of the external forcing factors (demersal fish harvesting rate, or river nutrient concentrations) was either doubled or halved. The difference between default and scenario states was measured by ΔX_i_ (eqn 28).

**Figure 6:**
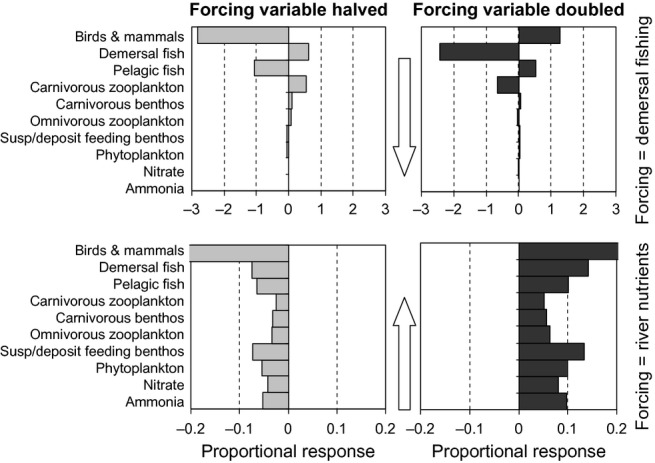
Proportional response in the stationary state annual average biomass of food web components in the North Sea food web model (Heath 2012), for (upper row) top-down forcing with half (left) and double (right) the default fishing pressure on demersal stocks, and (lower row) half and double river inputs of nitrate and ammonia. The proportional response is calculated as in eq. 28, i.e. log_2_(abundance with altered forcing factor/abundance with default forcing factor).

Doubling or halving of river nutrient concentration had a similar relative effect on all the functional groups in the food web, corresponding approximately to the archetypal bottom-up cascade (Fig.6). Top-down forcing by doubling or halving demersal fish harvesting rate, produced alternating positive and negative responses between lower functional groups but, due to compartmentalisation of the benthic and pelagic subsystems, this depended on how the groups were formed. Carnivorous zooplankton, that feed on omnivorous zooplankton (but also on fish larvae), changed together, as did carnivorous and sedimentary feeding benthos. However the dominant feature of the top-down forced profiles, was a strong attenuation of the response with decreasing trophic levels (Fig6 and Figs S4–S5 in Appendix S2).

## Discussion

### Density-dependent regulation of the end-members of a food chain is a key requirement for the generation of trophic cascades

Our first main finding was that the correlation signature of a top-down forced cascade–i.e. inverse correlations between adjacent pairs of trophic levels–was a robust feature of simple food chain formulations, i.e. independent of the form of uptake response or density dependence, provided that some form of density-dependent regulatory process was present at the lowest trophic level. Model variants which lacked a regulatory mechanism at the lowest trophic level exhibited ‘skipped-level’ responses to top-down forcing, i.e. positively correlated responses at alternate levels and no or weak response at intervening levels. Similarly, a positive response at all trophic levels to an increase in nutrient input rate occurred in all our cases where top-level density-dependent regulation was in place –but not where regulation was only at the lowest trophic level. In this case, a skipped-level response resulted from a lack of top-level regulation.

Our results explain the limited scope of earlier findings like Oksanen *et al*. (1981) where plant–herbivore–consumer food chain models with density-dependent regulation only of the plants showed the characteristic features of a top-down cascade in response to forcing of the consumers, but skipped-level responses to bottom-up forcing of the plants. The role of density dependence in the characteristics of cascades was recognised by McCann *et al*. (1998b) and Herendeen (2004), but the minimal requirement for regulation of the end-members of a food chain has not previously been highlighted.

### We show that cascade attenuation is related to the intensity of density-dependent mortality or uptake regulation, but there is an asymmetry in the attenuation of top-down and bottom-up cascades

We found that the details of cascade strength were sensitive to the nature of density-dependent regulation, especially in response to bottom-up forcing (Table3). On the basis of our results, we can account for the varied and sometimes conflicting outcomes of trophic cascade simulations reported by earlier investigators (e.g. Attayde & Ripa 2008; McCann *et al*. 1998b; Oksanen *et al*. 1981). Our mathematical analyses showed that both top-down and bottom-up forced effects could be amplified or attenuated with transfer between successive trophic levels depending on combinations of uptake responses and density-dependent regulation. Critically, we showed strong asymmetry between top-down and bottom-up forced cascades depending on the manner in which regulation was implemented. Both mortality and uptake regulation led to attenuation of top-down effects, i.e. reductions of top-down cascade strength. However, propagation of bottom-up cascades was more complicated. Herendeen (2004) concluded that the conditions for a strong bottom-up cascade are weak uptake regulation (ratio-dependent) and strong prey dependence of uptake rates, However, this conclusion was based on analysis of a 3-level food chain model which displayed clear skipped-level transmission between levels. Our results showed that skipped-level transmission is the default state of a bottom-up cascade in the absence of regulation except at the lowest trophic level. Increasing the intensity of mortality regulation damped out this between-level variability in responsiveness, whilst uptake regulation did not.

### We identify a need to distinguish between different types of regulatory processes in the formulation of ecosystem models

Our results showed that different forms of density dependence generate very different cascade dynamics in food chain models. From a modelling perspective, this indicates a need to see the various mathematical forms of density dependence not simply as devices to confer stability in population dynamics models, but as formal representations of particular biological processes. So, there is not necessarily one form which fits all situations, and different forms are not necessarily mutually exclusive. The issue is not the general primacy of consumptive vs. non-consumptive (density vs. trait-mediated) effects in nature or in models (Schmitz *et al*. 2004). Rather, it is the combination of such regulatory processes at different points in the system that permit model food webs to respond in different ways to both top-down and bottom-up forcing factors.

Three types of regulatory factors stand-out as requiring consideration in models. The first is cannibalism which clearly constitutes a density-dependent mortality in the context of individual species. However, the equivalent regulatory process in a model which only resolves guilds or functional groups implicitly includes predation between species. It is tacitly assumed that this can also be represented as a density-dependent mortality. The second is trait-mediated effects or predator avoidance behaviours induced by the fear of being eaten (Brown & Kotler 2004; Hammerschlag & Trussell 2011). These take a wide range of species-specific forms, the effects of which can be represented by consumer density-dependent regulation of species uptake rates. However, it is very unclear that equivalent processes act at the scale of species guilds. The third is largely unobserved agents such as disease organisms or parasites (Lafferty *et al*. 2008) which may also lead to density-dependent regulation of their hosts (Tompkins & Begon 1999). Explicit representation in food chain models will rarely be possible and then only in a species-specific context. However, density-dependent mortality may be a simple way to implicitly account for these missing regulatory effects.

Realistic representation of trophic cascades is a goal of most ecosystem models, and yet the sensitivity of simulated cascade properties to forms of density-dependent regulation has rarely if ever been taken into account. For example, the foraging arena formulation of consumer-density regulated uptake constitutes the universal basis of Ecosim, one of the more widely used marine food web models (Christensen & Walters 2004; Walters *et al*. 1997, 2000). On the basis of our findings regarding the behaviour of uptake regulation processes, we would predict that such a model would simulate archetypal top-down forced cascade dynamics, but tend to the skipped-level form of bottom-up cascade. In general, Ecosim models tend to show strong effects of predators on their prey, but not vice versa, i.e. harvesting of prey taxa has little effect on the productivity of their predators (Walters *et al*. 2005), apparently corroborating our prediction. In reality, there are innumerable instances of fisheries-forced changes in pelagic fish abundance, and nutrient-forced changes in primary production cascading up the food web to have noticeable effects on top-predators (e.g. Barber & Chavez 1983; Frederiksen *et al*. 2006, 2008).

### Implications for experimental and field observations of cascade effects

Our results point to a need for experimental studies to manipulate systems in such a way as to observe the effects of both top-down and bottom-up forcing. The asymmetry in the response to these two types of forcing is potentially useful in diagnosing the key mechanisms of internal regulation. We hypothesise that skipped-level effects in real-world or experimental systems should be diagnostic of either weak consumer regulation or a predominance of trait-mediated uptake regulation over density-mediated mortality regulation. However, interpreting the causes of cascade patterns and strength in natural systems is more difficult than in experimental systems, because top-down and bottom-up forcing factors are clearly not mutually exclusive in nature, and their effects will be conflated and combined with variations in any abiotic forcing effects such as dispersal rates (Rothschild 2011). Hence, we should expect conflicting results from analysis of field data relating cascade properties to, e.g. harvesting rates of upper trophic levels, especially if temporal trends in harvesting are correlated with trends in other forcing factors (Hunt & McKinnell 2006; Sala *et al*. 1998).

Our analysis suggests that some indices used to detect cascades in natural systems may give misleading results. Adhering to the archetypal view of cascade patterns, Frank *et al*. (2007) interpreted positive correlations between time-series of interacting pairs of trophic levels in a range of North Atlantic shelf sea food webs to be indicative of predominantly bottom-up forcing and, conversely, negative correlations to be indicative of top-down forcing. But, we show that weak or absence of correlation is not necessarily diagnostic of the type of forcing or cascade response. Skipped-level transmission in response to bottom-up forcing, results in uncorrelated variations in adjoining tropic levels in food chains that lack density-dependent regulation or perhaps are dominated by non-consumptive processes.

### Avenues for further investigation

In this study, we focused on the implications of alternative representations of density-dependent regulatory processes for cascade properties under two cases of functional relationship between per capita uptake rate and prey density (linear and Type-II). However, there is no reason to suppose that the biological processes represented by the different mathematical forms of density dependence should be mutually exclusive within any given food chain, or even for any given trophic component. Further analysis could determine how cascade properties are affected by interactions between different forms of density dependence, and between the parameters for density dependence and functional responses (Rossberg 2013). In addition, other functional forms for uptake rate have very different properties and potential consequences for cascades. The Holling Type-III relationship (eq. 3), e.g. can generate shifts between strong and weak predator–prey interactions in response to gradual changes in external forcing (top-down or bottom-up). When embedded in a food chain model, these cause switches between alternative stable regimes (Oguz & Gilbert 2007; Scheffer 2009) analogous to discontinuities predicted by network models of food webs in response to trends in interaction strength (McCann *et al*. 1998a). Examples in nature are restructuring of the marine food webs on the Scotian Shelf off eastern Canada (Frank *et al*. 2011) and in the Black Sea (Daskalov *et al*. 2007), both of which have coincidentally been cited as evidence of trophic cascades (Table1).

Finally, empirical data suggest a direct relationship between species diversity and the attenuation of trophic cascades (Baum & Worm 2009; Frank *et al*. 2007), yet the mechanisms remain unclear. Possibilities include scope for compartmentalisation and network connections with increasing species numbers (Krause *et al*. 2003). A key issue is how such effects could be reflected mathematically in food web models which cannot hope to explicitly represent the full range of species present in a system.
