# Supplementary material for: Understanding patterns and processes in models of trophic cascades
Source: Ecol Lett. 2013 Oct 27;17(1):101–14. doi: 10.1111/ele.12200 (PMC4237542; doi:10.1111/ele.12200)

Ecology Letters Supporting Information

Understanding patterns and processes in models of trophic cascades

Michael R. Heath[1], Douglas C. Speirs[1], John H. Steele[2]

[1]University of Strathclyde, Department of Mathematics and Statistics, Livingstone Tower, Glasgow, G1 1XP, UK. E-mail: m.heath@strath.ac.uk; d.c.speirs@strath.ac.uk
[2]Marine Policy Centre, Woods Hole Oceanographic Institution, Woods Hole, MA 02543, USA. E-mail: jsteele@whoi.edu

Appendix S1. Analytical steady state solutions to differential equations describing food chain model dynamics

Table S1. Analytical steady state solutions to equations 12 and 13 in the main text, for a Lotka Volterra system with density-dependent mortality regulation.

Rate equations	Steady state solutions for x_1 and x_2
$\frac{dx_1}{dt} = \sigma x_1 - x_1 x_2 - \lambda_1 x_1^2$	$x_1^* = \frac{(\delta + \lambda_2 \sigma)}{(1 + \lambda_1 \lambda_2)}$
$\frac{dx_2}{dt} = x_1 x_2 - \delta x_2 - \lambda_2 x_2^2$	$x_2^* = \frac{(\sigma - \lambda_1 \delta)}{(1 - \lambda_1 \lambda_2)}$

Table S2. Analytical steady state solutions to equations 14 and 15 in the main text, for a Lotka Volterra system with consumer density-dependent uptake regulation.

Rate equations	Steady state solutions for x_1 and x_2
$\frac{dx_1}{dt} = \sigma x_1 - \frac{x_1 x_2}{(\rho_2 + \upsilon_2 x_2)}$	$x_1^* = \frac{\delta \rho_2}{(1 - \sigma \upsilon_2)}$
$\frac{dx_2}{dt} = \frac{x_1 x_2}{(\rho_2 + \upsilon_2 x_2)} - \delta x_2$	$x_2^* = \frac{\sigma \rho_2}{(1 - \sigma \upsilon_2)}$

Table S3. Analytical steady state solutions to equations 16 - 18 in the main text, for a 3-level food chain (n=2) with a linear prey dependency of consumer uptake rate and top-level mortality regulation, with and without chemostat bottom-level regulation.

Rate equations	Steady state solutions for x_0, x_1 and x_2 without bottom-level regulation (φ = 0)	Steady state solutions for x_0, x_1 and x_2 with chemostat bottom-level regulation (φ = 1)
$\frac{dx_0}{dt} = \varphi(I - a_1 x_0 x)$	$x_0^* = x(0)$	$x_0^* = \frac{I}{a_1 x_1^*}$
$\frac{dx_1}{dt} = \varepsilon_1 a_1 x_0 x_1 - a_2 x_1 x_2$	$x_1^* = \frac{\delta + \lambda_2 x_2^*}{\varepsilon_2 a_2}$	$x_1^* = \frac{\varepsilon_1 I}{a_2 x_2^*}$
$\frac{dx_2}{dt} = \varepsilon_2 a_2 x_1 x_2 - \delta x_2 - \lambda_2 x_2^2$	$x_2^* = \frac{\varepsilon_1 a_1 x_0^*}{a_2}$	$x_2^* = \begin{cases} \frac{-\delta + \sqrt{\delta^2 + 4\varepsilon_1 \varepsilon_2 I \lambda_2}}{2\lambda_2} & \lambda_2 \neq 0 \\ \frac{\varepsilon_1 \varepsilon_2 I}{\delta} & \lambda_2 = 0 \end{cases}$

Table S4. Analytical steady state solutions to equations 19 - 21 in the main text, for a 3-level food chain (n=2) with consumer uptake rate determined by linear prey-dependency and consumer density-dependence, with and without chemostat bottom-level regulation.

Rate equations	Steady state solutions for x_0, x_1 and x_2 without bottom-level regulation (φ = 0)	Steady state solutions for x_0, x_1 and x_2 with chemostat bottom-level regulation (φ = 1)
$\frac{dx_0}{dt} = \varphi(I - a_1 x_0 x)$	$x_0^* = x(0)$	$x_0^* = \frac{I}{a_1 x_1^*}$
$\frac{dx_1}{dt} = \varepsilon_1 a_1 x_0 x_1 - \frac{a_2 x_1 x_2}{\rho_2 + x_2}$	$x_1^* = \frac{\delta x_2^*}{\varepsilon_1 \varepsilon_2 a_1 x_0^*}$	$x_1^* = \frac{\varepsilon_1 I}{a_2}\left(\frac{\rho_2}{x_2^*} + \upsilon_2\right)$
$\frac{dx_2}{dt} = \frac{\varepsilon_2 a_2 x_1 x_2}{\rho_2 + x_2} - \delta x_2$	$x_2^* = \frac{\varepsilon_1 a_1 x_0^* \rho_2}{a_2 - e_1 a_1 x_0^* \upsilon_2}$	$x_2^* = \frac{\varepsilon_1 \varepsilon_2 I}{\delta}$

Table S5. Analytical steady state solutions to equations 22 - 24 in the main text, for a 3-level food chain (n=2) with a Type-II prey-dependency and top-level mortality regulation, with and without chemostat bottom-level regulation

Rate equations	Steady state solutions for x_0, x_1 and x_2 without bottom-level regulation ($\varphi = 0$)	Steady state solutions for x_0, x_1 and x_2 with chemostat bottom-level regulation ($\varphi = 1$)
$\frac{dx_0}{dt} = \varphi\left(I - \frac{a_1 x_0 x_1}{k_0 + x_0}\right)$	$x_0^* = x(0)$	$x_0^* = \frac{I k_0}{\left(a_1 x_1^* - I\right)}$
$\frac{dx_1}{dt} = \frac{\varepsilon_1 a_1 x_0 x_1}{k_0 + x_0} - \frac{a_2 x_1 x_2}{k_1 + x_1}$	$x_1^* = \frac{a_2 x_2^* (k_0 + x_0^*)}{\varepsilon_1 a_1 x_0^*} - k_1$	$x_1^* = \frac{\varepsilon_1 k_1 I}{\left(a_2 x_2^* - \varepsilon_1 I\right)}$
$\frac{dx_2}{dt} = \frac{\varepsilon_2 a_2 x_1 x_2}{k_1 + x_1} - \delta x_2 - \lambda_2 x_2^2$	$x_2^* = \frac{(\varepsilon_2 a_2 - \delta) \pm \sqrt{(\delta - \varepsilon_2 a_2)^2 + \frac{4\lambda_2 \varepsilon_2 \varepsilon_1 a_1 k_1 x_0^*}{(k_0 + x_0^*)}}}{2\lambda_2}$	$x_2^* = \begin{cases} \frac{-\delta + \sqrt{\delta^2 + 4\varepsilon_1 \varepsilon_2 I \lambda_2}}{2\lambda_2} & \lambda \neq \text{[illegible]} \\ \frac{\varepsilon_1 \varepsilon_2 I}{\delta} & \lambda = 0 \end{cases}$

Table S6. Analytical steady state solutions to equations 25 - 27 in the main text, for a 3-level food chain (n=2) with consumer density-dependent non-linear (Type-II) prey-dependency, with and without chemostat bottom-level regulation.

Rate equations	Steady state solutions for x_0, x_1 and x_2 without chemostat bottom-level regulation ($\varphi = 0$)	Steady state solutions for x_0, x_1 and x_2 with chemostat bottom-level regulation ($\varphi = 1$)
$\frac{dx_0}{dt} = \varphi\left(I - \frac{a_1 x_0 x_1}{k_0 + x_0}\right)$	$x_0^* = x(0)$	$x_0^* = \frac{I k_0}{\left(a_1 x_1^* - I\right)}$
$\frac{dx_1}{dt} = \frac{\varepsilon_1 a_1 x_0 x_1}{k_0 + x_0} - \frac{a_2 x_1 x_2}{k_1 + x_1 + \upsilon_2 x_2}$	$x_1^* = \frac{\delta x_2 (k_0 + x_0^*)}{\varepsilon_1 \varepsilon_2 a_1 x_0^*}$	$x_1^* = \frac{\varepsilon_1 I\left(k_1 + \rho_2 x_2^*\right)}{\left(a_2 x_2^* - \varepsilon_1 I\right)}$
$\frac{dx_2}{dt} = \frac{\varepsilon_2 a_2 x_1 x_2}{k_1 + x_1 + \upsilon_2 x_2} - \delta x_2$	$x_2^* = \frac{\varepsilon_1 \varepsilon_2 a_1 k_1 x_0^*}{\varepsilon_2\left(a_2 k_2 + a_2 x_0^* - \varepsilon_1 a_1 \rho_2 x_0^*\right) - \delta\left(k_0 + x_0^* \right.}$	$x_2^* = \frac{\varepsilon_1 \varepsilon_2 I}{\delta}$

Figure S1. Analytical steady states of a simple food chain system with non-linear Type-II prey-dependency (equations 22-27), to top-down (TD) and bottom-up (BU) forcing, depending on whether regulation is located at the bottom-level (BL), top-level (TL) or both, of the food chain. Parameters used in the analysis were $a_i = 1$; $\varepsilon_i = 1$, $\delta = 0.1$ (except when treated as the driving variable), $\lambda = 0.1$, $\upsilon = 0.1$. Solid lines for cases 2, 3, 5 & 6 show the response with top-level mortality regulation, dashed with top-level uptake regulation.

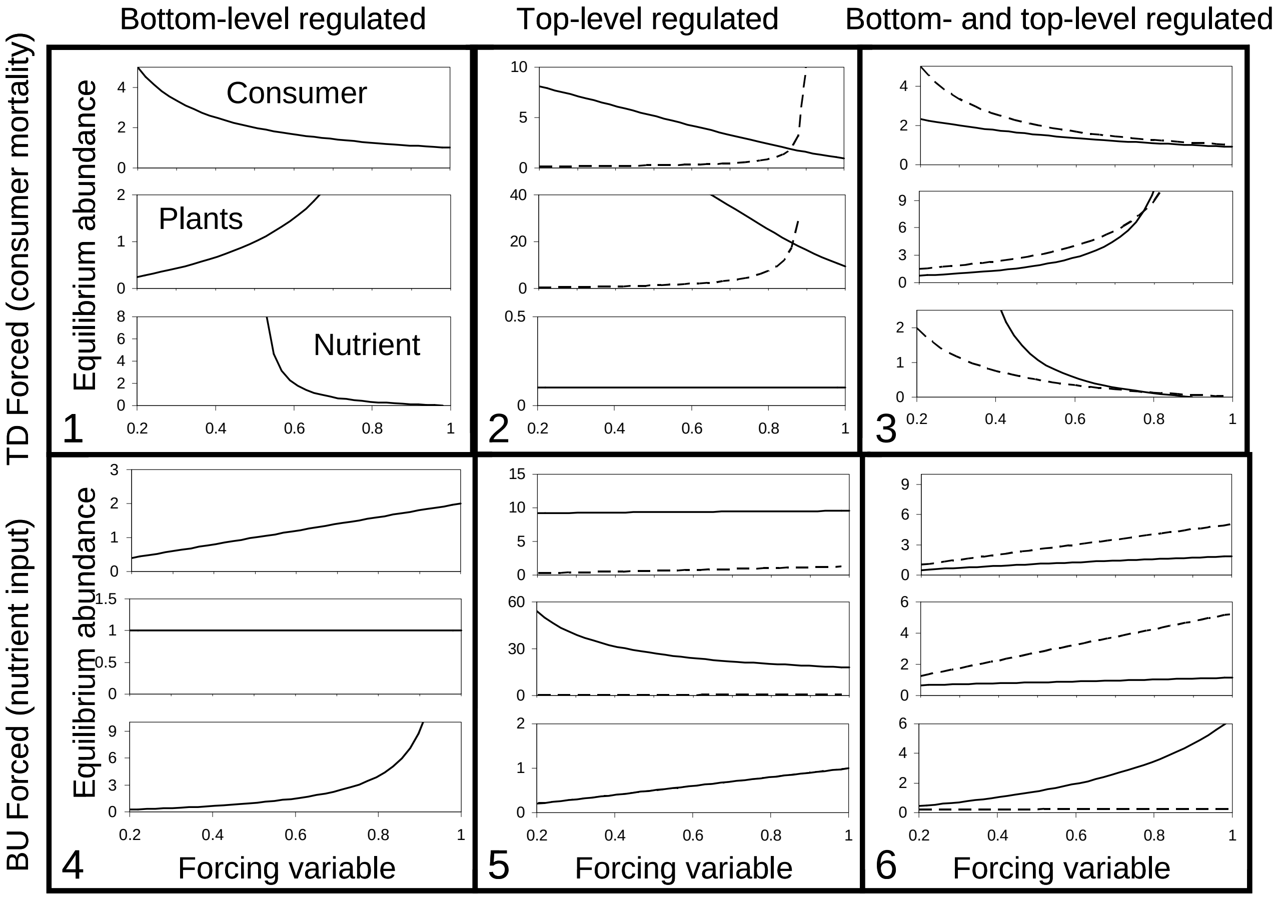

Figure S2. Proportional response of the 4-level food chain models to a doubling of top-down forcing (density independent mortality; top row), or bottom-up forcing (nutrient input rate; bottom row), with mortality or uptake regulation at the top and interior-trophic levels, i.e. as Fig.5 in the main manuscript, except with regulation at interior as well as at the top ($\lambda_i = 1$, $\upsilon_i = 1$, i = 1 to n). Left column, linear prey dependency; right column, Type-II non-linear prey dependency. Pale shaded bars, uptake regulation; dark shaded, mortality regulation. The proportional response is calculated as in eq. 28 of the main text, i.e. $\log_2$(abundance with altered forcing/abundance with default forcing).

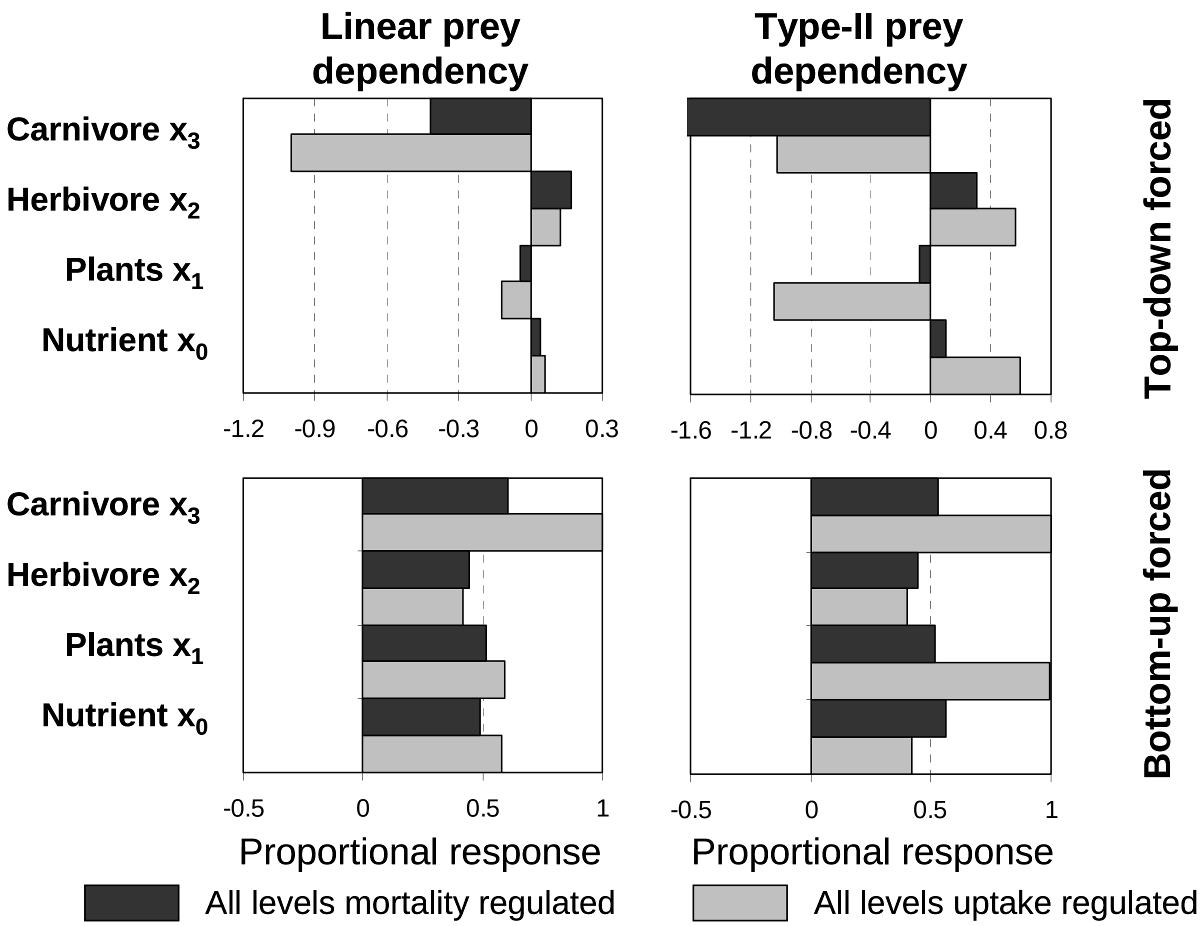

Supplement: Supplementary file 2 — Supplementary [file ele0017-0101-SD2.pdf]
