# Supplementary material for: Understanding patterns and processes in models of trophic cascades
Source: Ecol Lett. 2013 Oct 27;17(1):101–14. doi: 10.1111/ele.12200 (PMC4237542; doi:10.1111/ele.12200)

Ecology Letters Supporting Information

Understanding patterns and processes in models of trophic cascades

Michael R. Heath[1], Douglas C. Speirs[1], John H. Steele[2]

[1]University of Strathclyde, Department of Mathematics and Statistics, Livingstone Tower, Glasgow, G1 1XP, UK. E-mail: m.heath@strath.ac.uk; d.c.speirs@strath.ac.uk
[2]Marine Policy Centre, Woods Hole Oceanographic Institution, Woods Hole, MA 02543, USA. E-mail: jsteele@whoi.edu

Appendix S2. North Sea food web model

2.1 Model summary description (reproduced from Heath (2012))

The model simulated fluxes of a single nutrient element (nitrogen), between bulk mass state variables representing classes of detritus, dissolved nutrient, phytoplankton, benthos, zooplankton, fish, and top-predators. The state variables were further resolved to represent two depth layers in the water column, and a seabed sediment layer (Fig. S3). Rates of exchange between the mass compartments were described by a set of ordinary differential equations. Key features of the model were:

- External sources of nitrogen were from phytoplankton, detritus, nitrate and ammonia advected into the model domain by ocean currents, atmospheric deposition and river inputs of nitrate and ammonia.
- Exports of nitrogen from the model (sinks) were phytoplankton, detritus, nitrate and ammonia advected out of the model domain, nitrogen gas produced by denitrification, and fishery landings.
- Phytoplankton, detritus, nitrate and ammonia in the water column were subject to vertical exchange between depth layers (by sinking of particulate material and mixing). Detritus, ammonia and nitrate were subject to vertical exchange between the water column and sediment.
- Uptake of dissolved ammonia and nitrate by phytoplankton was confined to the surface layer and modeled according to a Type–II function scaled by the depth averaged daily irradiance. Maximum uptake rate was temperature dependent according to a Q_{10} function, but the half-saturation coefficient was temperature independent.
- A constant proportion of phytoplankton per day was converted to detritus.
- Uptake of prey by all classes of predators was described by Type-II functions with prey and temperature-dependent maximum uptake rates. The half-saturation coefficient was independent of both temperature and prey.
- Fixed proportions of food ingested by predators were assimilated, excreted to ammonia, and defaecated to detritus.
- Predators excreted a temperature-dependent proportion of their body mass per day to ammonia, according to a Q_{10} function.
- Predators were subjected to a density-dependent mortality rate, which created a flux to corpses.

- A proportion of corpse mass was converted to detritus per day, and similarly detritus to ammonia, ammonia to nitrate, and nitrate to nitrogen gas (denitrification). The proportions were temperature dependent according to a Q_{10} function.
- Fish were resolved into two demographic stages – larvae (including eggs), and adults. Adults shed a fixed proportion of their mass per day to larvae during prescribed time intervals each year. A fixed proportion of larvae were promoted to adults per day during a different prescribed interval.
- Adult fish and benthos categories were subject to harvesting which removed a proportion of their mass per day as catch. A fraction of the catch was returned to the food web as fishery discards, the remainder was regarded as landings which were a sink.
- Benthos categories additionally suffered a by-catch mortality which was a fixed fraction of the demersal fish harvesting rate. This by-catch was passed directly to fishery discards.
- The proportion of catch discarded was constant for pelagic fish and benthos, but scaled with adult abundance for demersal fish, to caricature the shift in fish size distribution towards smaller individuals with declining abundance in demersal fish communities.

A full technical description of the model is given in Heath (2012). The model was implemented in the R statistical environment version 2.11.1 (R Development Core Team 2005), and used the lsoda routine in the package odesolve to solve the differential equations and output values of the state variables and fluxes at daily time intervals.

2.2 Default model run

Model parameters were optimized by simulated annealing to identify the set providing the best fit of the stationary state model to a suite of observations on biomasses and fluxes in North Sea averaged over the period 1970-1999, whilst being driven by a repeating annual cycle of 1970-1999 monthly averaged driving variables. We refer to this as the 'default model run', and averaged the state variable abundances over a stationary annual cycle.

2.3 Scenario model runs

We defined 4 scenario runs of the model based on halving or doubling of various external driving time series: a) halving or doubling of the default nitrate and ammonia concentrations in inflowing river waters; b). halving or doubling of the default harvesting rate applied to demersal fish. For each model state variable X (averaged over a stationary annual cycle), the difference between default and scenario states was expressed as $\Delta X = \log_2(X_{scenario}/X_{default})$. Hence $\Delta X = 0$ corresponds to no difference between scenario and default, $\Delta X = 1$ to a doubling of abundance in the scenario run, and $\Delta X = -1$ to a halving.

2.4 Comparison of scenario and default model results

The results showed several key features:

1) Halving and doubling of demersal harvesting rates led to inverse responses throughout the water column food web (Fig.S4). However, this was not universally the case in the recycling food web, with water column ammonia, corpses, and carnivorous/scavenging benthos all showing a positive response to both halving and doubling of demersal harvesting rate. This occurred because corpses, which constituted a significant portion of the diet of carnivorous/scavenging benthos, were produced by a combination of fishery discards, and quadratic density dependent mortality terms applied to upper trophic components of the food web. Corpse

production showed a U-shaped response to demersal harvesting rates (Heath 2012). Carnivorous/scavenging benthos abundance was close to a local minimum with respect to fish harvesting rates in the default model run.

2) The conceptual top-down alternating responses were evident along some trophic pathways, e.g. demersal fish, pelagic fish, omnivorous zooplankton, phytoplankton, nitrate. However, carnivorous zooplankton were anomalous in this respect, always responding in the same direction as their main prey (omnivorous zooplankton). In the model, carnivorous zooplankton were both predators on fish larvae (pelagic and demersal), and prey of adult fish. Hence the connectivity of carnivorous zooplankton in the food web was complex.
3) Changes in demersal fish landings responded negatively to both a doubling and halving of the harvesting rate. This was because landing under the default harvesting rate were close to the maximum sustainable yield, so any change in harvesting rate was guaranteed to produce a decrease in landings. Conversely, pelagic landings responded positively and negatively to a doubling and halving of demersal harvesting rate respectively due to connectivity between demersal and pelagic fish in the food web.
4) In marked contrast to the patterns above, doubling of river nutrient concentrations produced an increase in stationary annual average abundances throughout the entire web (Fig. S5). Conversely, halving river nutrients produced a decrease throughout the web.

References

Heath, M.R. (2012). Ecosystem limits to food web fluxes and fisheries yields in the North Sea simulated with an end-to-end food web model. *Prog. Oceanogr.*, 102, 42-66.

Figure S3 Schematic showing the North Sea food web model components and driving variables, in relation to physical structures (surface water layer, deep water layer, and sediments).

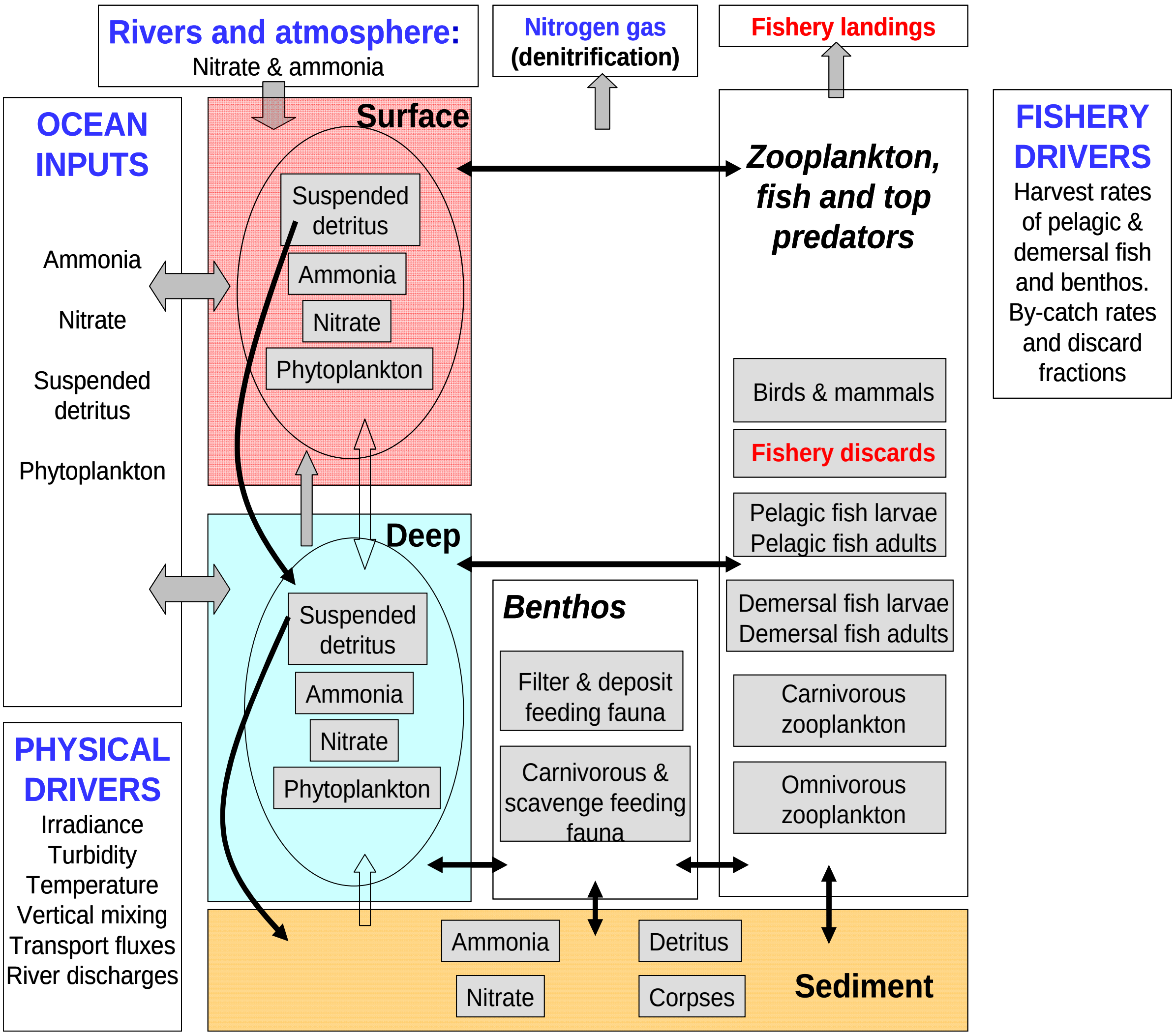

Figure S4 Changes in stationary annual average abundances of ecosystem components in the North Sea food web model parameterized against observations collected during 1970-1999 (Heath, 2012), as a result of doubling (left) and halving (right) the demersal (benthivorous and piscivorous) fish harvesting rate. Numerical values of the change are indicated by a $\log_2$ scale, so that +1 represents a doubling of abundance relative to the 1970-1999 model, and -1 indicates a halving of abundance. Boxes with dashed outlines represent fishery landings and hence export fluxes out of the system. Black arrows indicate uptake fluxes, green arrows indicate fishery harvests. Fluxes due to excretion and death are not shown for clarity.

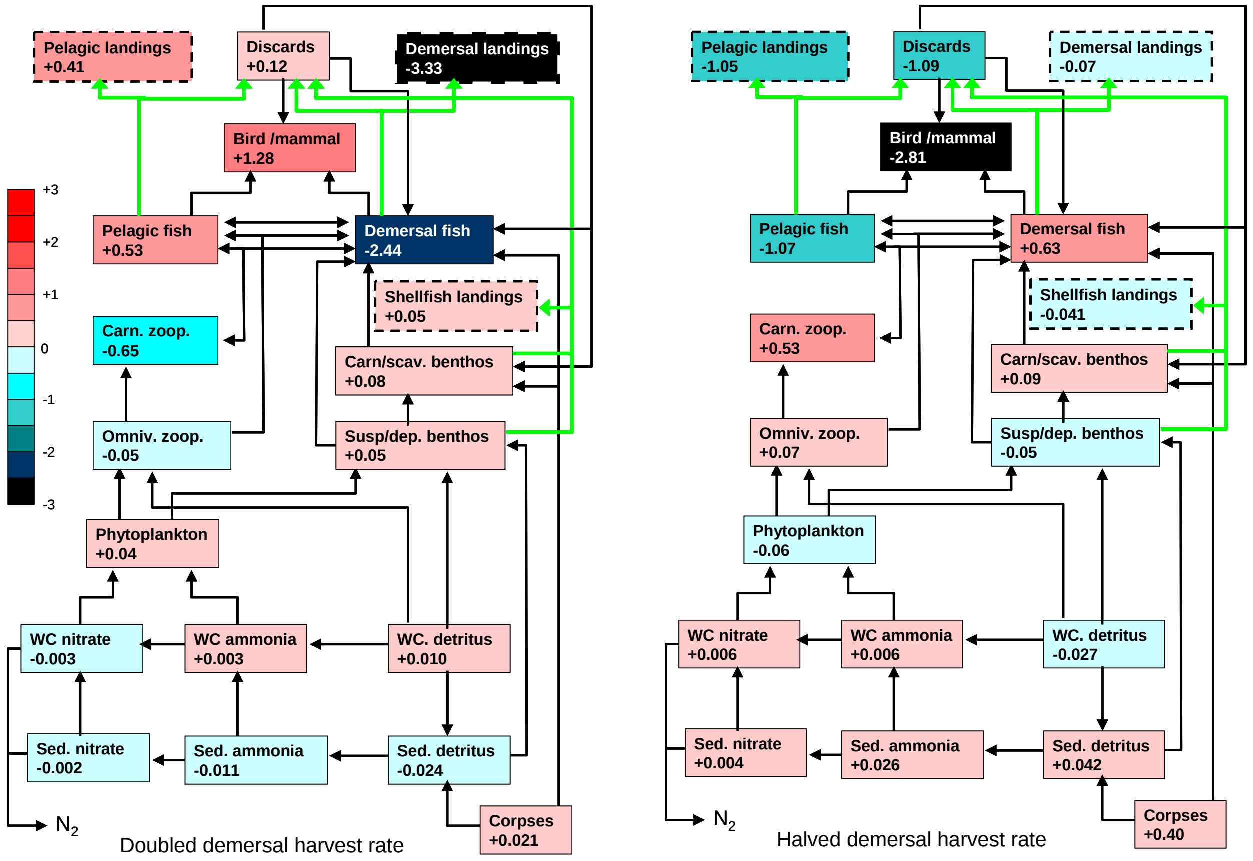

Figure S5 As Fig. S4 but showing the response to doubling and halving the concentrations of nitrate and ammonia in rivers flowing into the North Sea.

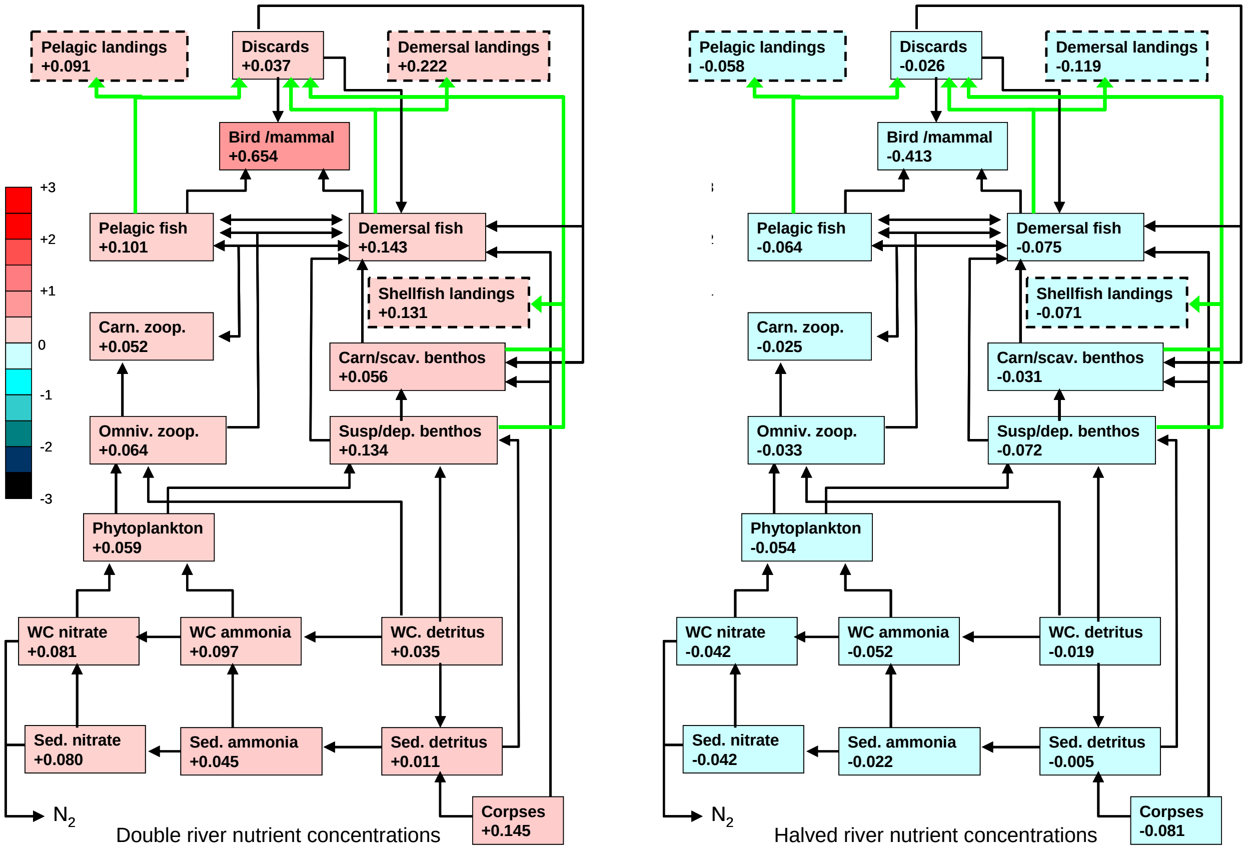

Supplement: Supplementary file 4 — Supplementary [file ele0017-0101-SD4.pdf]
